# Innovative technologies for removal of micro plastic: A review of recent advances

**DOI:** 10.1016/j.heliyon.2024.e25883

**Published:** 2024-02-10

**Authors:** Muhammad Salman Nasir, Ifrah Tahir, Ahsan Ali, Iqra Ayub, Abdul Nasir, Naseem Abbas, Uzair Sajjad, Khalid Hamid

**Affiliations:** a Department of Structures and Environmental Engineering, University of Agriculture, Faisalabad, 38040, Pakistan; b Guangdong Technion-Israel Institute of Technology, Shantou, 515063, China; c Department of Energy Systems Engineering, University of Agriculture, Faisalabad, 38040, Pakistan; d Department of Mechanical Engineering, Sejong University, Seoul, 05006, South Korea; e Department of Energy and Refrigerating Air-Conditioning Engineering, National Taipei University of Technology, Taipei, 10608, Taiwan; f Process and Power Research Group, Department of Energy and Process Engineering, Norwegian University of Science and Technology (NTNU), Trondheim, Norway

**Keywords:** Microplastic, Wastewater, Removal, Polymers, Pollution

## Abstract

Plastics are becoming a pervasive pollutant in every environmental matrix, particularly in the aquatic environment. Due to increased plastic usage and its impact on human and aquatic life, microplastic (MP) pollution has been studied extensively as a global issue. The production of MP has been linked to both consumer and commercial practices. There is a significant amount of MP's that must be removed by wastewater treatment plants before they can be bioaccumulated. Many researchers have recently become interested in the possibility of eliminating MPs in wastewater treatment plants (WWTP). Many studies have analyzed MP's environmental effects, including its emission sources, distribution, and impact on the surrounding environment. The effectiveness of their removal by various wastewater treatment technologies requires a critical review that accounts for all these methods. In this review, we have covered the most useful technologies for the removal of MP during WWTP. The findings of this review should help scientists and policymakers move forward with studies, prototypes, and proposals for significant remediation impact on water quality.

## 1 Introduction

Plastics are becoming a persistent pollutant in every environmental matrix, particularly in the aquatic environment. Plastic manufacturing has increased to 348 million tons worldwide. Plastics typically last hundreds of years in the environment in natural settings due to their sturdy architecture. Plastics, on the other hand, disintegrate into smaller fractions termed primary and secondary microplastics (MPs) because of UV radiation, weathering processes, and environmental aging [1]. Primary MPs are produced during industrial manufacturing, while secondary is their sub-derivatives in form of polymers that are produced during biological, physical, or chemical degradation [2]. The issue of MP pollution has recently gained attention worldwide because to the extensive use of plastics in various human activities, as well as the discharge of untreated home and industrial wastewater. Scientific evidence has demonstrated that untreated microplastics are frequently released from wastewater treatment plants (WWTPs), enter bodies of water, and ultimately accumulate in the environment. Hence, it is important to investigate the efficacy of various treatment methods in wastewater treatment plants (WWTPs) for addressing the issue of microplastics. Additionally, comprehending the process behind the removal of microplastics is crucial in order to mitigate their entry into the natural aquatic ecosystem. However, in this review we will try to summarize the microplastic removal treatment technologies in the WWTPs.

A major amount of plastic comes from inland sources as an artificial product and up to 80% end up in the oceans because of their release through the home and industrial wastewater, wind transport, and surface run-off. This inclusion in the ocean with time will contribute to nano plastics and their derivatives. Plastics undergo immediate changes in their characteristics upon exposure to environmental weathering processes [3]. However, the presence of processes such as hetero aggregation with other detritus and suspended particles might make these materials susceptible to sedimentation in riverbeds.

The first mass production of plastics begins in the 1950s [4] Since then, manufacturing has increased to meet global needs of over 348 million tons and is expected to quadruple by 2035 [5]. According to the United Nations Environment Program (UNEP), marine litter includes over 80% of plastic waste, and projection shows the ocean will contain more trash than fish. It takes hundreds of years for the complete degradation of polymers. They are considered a dangerous form of pollution due to their accumulative and persistent features.

Plastics are an extensive class of synthetic materials composed of over 5000 different grades of polymers and other chemicals. Polymers with a diameter of under 5 mm (mm) are referred to as MPs. However, some investigations have classified MPs as particles measuring less than 1 mm, 2 mm, 2–6 mm, and 10 mm [6]. All over the world, MPs were discovered in freshwater bodies, the arctic ice, beaches, subtidal and deep-sea sediment, and the water column [7,8]. MPs infiltrate water systems by a variety of routes, including overland flow, wind convection, and WWTP discharge. They attracted more attention because of their deleterious effects on aquatic microorganisms like *Mytilus edulis*. Direct consumption of MPs by aquatic species results in central impairment, and reproductive issues while it also impacts human health as the part of the food chain [9].

Studies have focused on the marine environment since the twenty-first century begins [10,11]. However, in recent years, the relevance of MPs in freshwater has gained attention. The increasing concern over plastic particles (PPs) in the millimeter size range stems from their detrimental impact on environmental quality preservation and associated matters. MPs on the other hand have been discovered to be much more prevalent in coastal areas than in offshore regions which might be ascribed to anthropogenic activities, such as in densely inhabited areas with heavy industrial and commercial activity [12]. Migration between seawater and seafloor sediments is primarily influenced by MPs density. In the marine environment, density is 0.9–1.4 g/cm3 with a variety of colors and shapes. Furthermore, they have the properties such as density, shape, and size similar to larger minerals which alter transportation [13]. As a result, MP's movement and distribution patterns in the marine environment are undifferentiable to those of natural organic matter and minerals.

Research has demonstrated that microplastic trash in aquatic environments exhibits the capacity to absorb and subsequently release chemicals that provide a potential threat to the well-being of aquatic organisms [11]. Moreover, studies have demonstrated the ability of these compounds to be transferred to higher trophic levels within the food chain, hence possibly impacting the well-being of animals across all levels of the ecosystem [18]. Furthermore, it has been observed that microplastics have a significant impact on the behavior of aquatic organisms, leading to modifications in their eating patterns and decreased reproductive achievements [[19], [20], [21], [22]]. Microplastics have been detected in the gastrointestinal tracts and excrement of various terrestrial fauna, encompassing avian species, small mammals, and insects. Previous studies have demonstrated that the consumption of microplastics has deleterious effects on the well-being of these organisms, leading to a decline in their physical state and disruption of their immune system processes [[23], [24], [25]]. Microplastics' effects on animals are not limited to marine and terrestrial ecosystems. The presence of micro plastics in the atmosphere has also been documented, and the complete understanding of the possible effects of airborne microplastics on animals remains incomplete [[26], [27], [28]].

Many questions remain unanswered about the environmental distribution, destiny, and movement of microplastics, despite the rising corpus of studies on their presence and possible implications on animals. According to estimates, if the emission rate is not promptly reduced, the amount of microplastic pollution in the global oceans is projected to increase by over two-fold, reaching 3 Mt annually by the year 2040 [29]. Considering the widely accepted range of environmental lifetimes spanning from thousands to billions of years, the escalating rate of microplastics production has led to the emergence of a significant concern over the impact of microplastic pollution on the overall health landscape.

MPs, like other toxic elements, are produced by a variety of land-based sources and ultimately find their way to WWTPs, which are thought to serve as the link between man-made toxins and their natural environments [14]. A dominant route of MPs passivation in an aquatic ecosystem is through WWTP effluents [15]. These facilities collect wastewater from social economic zones to treatment plants so that it can be recycled and released back into the environment to contain the damage. WWTPs were not intended to remove MPs from wastewater, it is expected that after depuration MPs are still present and deposited in the sludge. The removal effectiveness varies between 64 and 99% [16].

WWTPs are widely believed to be the major exchanger of MPs from land-based sources; because they transform primary PPs into secondary MPs [15,17]. These particles also mix with sludge instead of WWTPs released water, which later becomes a part of agricultural soil [18]. But by air condensation, MPs from the environment that are produced by plastics producers and automobiles get up in WWTPs. These plants consistently discharge untreated MPs into the environment, where they eventually end up polluting water systems and accumulating in the ecosystem [19]. Therefore, to decrease the number of MPs that enter the natural aquatic environment, it is essential to study the impact of MPs in WWTPs employing various treatment methods and to understand the process of microplastic removal. Additionally, there is a dearth of research describing in detail the methods used by major WWTP treatment systems to filter out MPs.

Earlier research on WWTPs found that the MPs which were not completely removed from the wastewater were reduced by 99% through post-physical, chemical, and biological treatment [20]. Different WWTPs used the same strategies with varying degrees of success in removing the MPs. For instance, at a Beijing WWTP, MPs were eliminated by employing air circulation coarse chambers, anaerobic-anoxic (A2O), and advanced oxidation (UV and O3) processes, with respective removal efficiencies of 58.84%, 54.47%, and 71.67%. MPs removal efficiencies of 49.56%, 26.01%, and 78% were achieved using the same treatment processes in a Shanghai WWTP [21]. These results imply that it is very challenging to understand the effect of a specific remediation technique on MPs elimination in a WWTP through a solitary experiment. Moreover, qualitative rather than quantitative analyses have been the standard in the past when studying methods for eliminating MPs. Therefore, new methods must be developed to evaluate the efficacy WWTPs.

### 1.1 Occurrence of micro-plastics in wastewater

The most common forms of MPs are fibers and microbeads. Microbeads, with an average size of <5 mm, are commonly found in cosmetic products that are released through washing.

It was reported that every time uses an exfoliant or washing cosmetics you release one microbead or particle per liter [17] However, synthetic fibers release about 34.8% of their MPs into the water when washed [22]. The majority of the MPs come from nonresidential sources, specifically air blasting, manufacturing, transportation, Styrofoam products, the textile industry, and debris from excavating and trimming plastics [23].

**Fig. 1:**
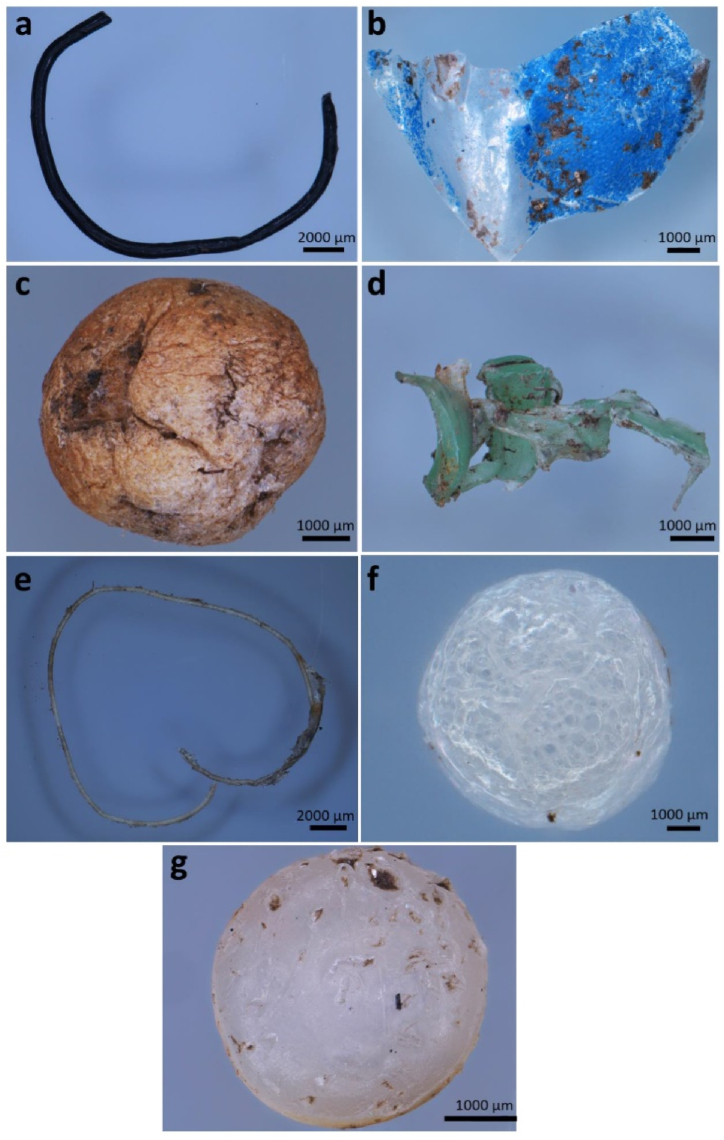
Different forms of microplastics (a) Fiber; (b) film; (c) foam; (d) fragment; (e) monofilament line; (f) microbead; and (g) pellet. Reprinted with permission from [40].

According to reports, temperature and exposure to solar radiation are conducive to the production of MPs. In contrast, frozen and anaerobic environments slow down the pace at which PPs degrade [24]. The most well-known types of MPs include (PE) polyethylene, (PP) polypropylene, (PVC) polyvinyl chloride, (PA) polyamide, (PET) polyethylene terephthalate, (PS) polystyrene, and (PC) polycarbonate [25]. Depending on their origin and source, MPs can take on a variety of forms, such as fibers, pieces, pellets or beads, adhesive, foil, etc. as shown in Fig. 1(a–g).

(a) Fibers

One of the most common types of MP pollution found in water is microplastic fibers (MF). Water treatment plant effluents have been identified as a particularly significant contributor [26]. There is a wide variety of MPs in the marine environment, but evidence from around the world suggests that in many places, anthropogenic fibers make up the vast majority of marine MPs (up to 91%) [27].

Some cellulose fibers that have gone through industrial processing and may contain dyes or other synthetic chemicals could also be included here [27]. Polyester is by far the most common polymer used in synthetic fibers, but other polymers like polyamide, polyacrylic, polyethylene, and polypropylene are also used [28]. So, like other MPs, MFs are all over the place and resistant to decomposition [29]. However, the current hydraulic retention duration in WWTPs (7–14 h) is insufficient for efficient microplastic breakdown. Their elongated structure means that their physical properties are distinct from those of other MPs, which may affect how they form, sink, move laterally, and degrade.

In terms of abundance in the affluent, fiber often comes in first, followed by pieces, pellets, and beads. According to Gies et al. (2018) [30], a significant proportion of MPs discovered in primary and secondary leachate wastewater samples (65.6%) were fibers, accompanied by particles (28.1%) and microbeads (5.4%). In contrast, only a tiny portion of the influent was made up of granules, foam, and sheets (0.45, 0.22, and 0.20%, respectively). That's also consistent with the findings of Liu et al. (2019) on the (SAMPs) suspended atmospheric microplastic, who discovered that microfiber accounted for 67% of all SAMPs, while granules and fragments accounted for 3% and 30% respectively. This suggests a strong connection between the polluting of other habitats by MP air fallout [31].

(b) Fragments

Even while some research [32] asserted that fragments predominate in the effluent, fragments typically rank inferior to sludge predominance [33]. Cumulative drainage and displaced (stormwater) are linked to an increase in fragment and foam particle concentration in effluent, respectively [34]. Variations in values have been discovered among 7 Wastewater treatment plants that release water into Xiamen Bay. Granules (41.1%) and fragments (31.3%) were the two most common shapes of MPs, followed by fibers (23.7%) and pellets (3.9%) [35].

According to research by Mason et al., whereas bigger particles were predominately made up of fibers, tiny granules were evenly distributed between fragments and fibers (80%) [34]. The considerable quantity of flakes and fragments indicates the importance of secondary MPs and attempts to reduce MP removal into the atmosphere must concentrate on eradicating these pollutants from their sources [36].

(c) Microbeads

Most microbeads originate from cosmetics and products of personal care for consumers, which account for 6–7 % of the outcome (mostly scrubs). Healthcare products have the smallest average particle size and more than 3000 particles per gram [37]. According to Napper et al. using skincare products like face washes could discharge 4594 and 94,500 microfibers after just one use [38]. Small variations in microbead size and color between brands suggest that manufacturers may adhere to a common standard [39]. Polyethylene particles are present in well over 80% of foot cleanses and 60% of body cleanses adhere to a common standard [37].

## 2 Removal of microplastics in the WWTPs

The majority of MPs found in wastewater treatment plants come from urban activities (tire damage, paint shedding, plastic/textile industries) and domestic items (cosmetics, exfoliants, scrubbers, and textiles) [41]. The regular use of microfibers in fabrics could even cause them to release into the air, making them a significant source of MPs in the atmosphere [42]. MPs suspended in the atmosphere may re-accumulate on land, in rivers, and in the oceans through wet or dry deposition [43]. Since land deterioration can result in the transport of more plastic debris, rainfall has a significant impact on MPs relocation [44]. As a result, while assessing MP routes to aquatic habitats, unmanaged plastic debris should be considered. Garbage was discovered to be a potential origin rather than a sink for aquatic MPs. Plastic films, soil conditioners, and organic fertilizers have been identified as the primary MP sources for soil contamination [45]. During the skimming and settling operations, wastewater deposits from WWTPs transport MPs subsequential as an alternative to organic fertilizers [34].

**Fig. 2:**
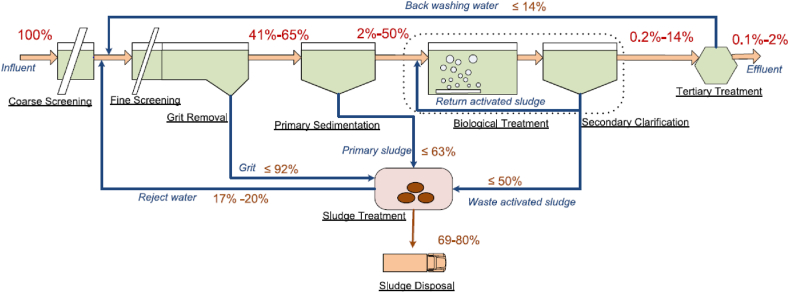
The estimated particle flow of microplastic in WWTPs after the primary, secondary and tertiary treatments. Reprinted with permission from Ref. [15].

Various techniques are there to treat MPs mainly wastewater treatment plants which are used to treat MPs. Individual field samples taken from the primary treatment processes, the secondary, and the tertiary were used in previous research to determine MPs removal efficiency. The removal efficiency of the WWTPs is mainly based on the amount of influent and effluent discharge. Fig. 2 shows the extraction rate of primary, secondary, and tertiary treatments based on different literature [15,46]. However, these types of research were unable to pinpoint the best treatment method and mechanism for removing MPs. WWTPs employed a wide range of primary, secondary, and tertiary treatment technologies, such as grease and grit treatment, primary settling, A2O, biofilters, and other bioreactors, as well as biologically active filters (BAFs), disc filters (DFs), and rapid sand filters (RSFs).

### 2.1 Preliminary treatment

Preliminary and primary treatment can remove efficiently the bulk of the MPs in wastewater. According to the study, between 35% and 59 % of the MPs may have been eliminated during the initial treatment, and between 50% and 98 % may have been separated after the initial treatment [21]. The main removal techniques at this point involved slotting the heavy suspended microplastic during grease or surface skimming on the primary clarifiers as well as skimming the light suspended microplastic during grit disposal and gravity separation in primary clarifiers. Due to its ability to successfully remove larger MPs, pre-treatment had the biggest influence on the distribution of microplastic size. The fraction of large molecules (1000 mm–5000 mm) fell from 45% to 7% following the first intervention, according to Dris et al. (2015) [47]. According to studies on the morphologies of Mps, pre-treatment can more effectively remove fibers from wastewater than fragments; also, following pre-treatment, the relative abundance of fibers is reduced [48,49].

This might be explained by the fact that fibers are collected more readily in emulsification particles and separated by deposition. According to Michielssen et al. [50], microbeads were not found in the effluent of wastewater treatment plants. A survey of WWTPs in New York, USA, indicated that four out of every ten still discharge microbeads. This disparity could be attributed to differences in the number of fats, lubricants, and oil in the effluent, because these substances may favor the removal of MPs.

#### 2.1.1 Grit chamber/primary sedimentation

The grit chamber is located after the screen and before the primary sedimentation tank, functioning as the second step in the primary treatment process. Its purpose is to effectively eliminate sand and grit particles from the wastewater. The inclusion of a grit chamber is a fundamental component within the infrastructure of a municipal wastewater treatment facility. Typically, the addition of a grit chamber is optional in industrial wastewater treatment facilities. The purpose of this design is to effectively eliminate inorganic particles that possess a specific gravity of approximately 2.65 and a minimum particle diameter for removal of 0.20 mm. In practical applications, it is not uncommon for a grit chamber to be specifically engineered to effectively eliminate particles of minimal size, often measuring 0.15 mm. The particles that make up grit can be anything from sand and gravel to cinder or any other dense material with a specific gravity higher than that of living organisms. The positioning and arrangement of the grit chamber play a crucial role in protecting the pumps and other equipment from abrasion and wear. If the sewage treatment plant doesn't include a grit chamber, there will be more equipment maintenance, grit will settle into pipes, channels, and conduits, and the cleaning interval of the digester will be shorter because inert elements will build up in the digested sludge more quickly.

The initial stage of the wastewater treatment process consists of grit formation and primary deposition. MPs are mostly eliminated at this initial phase of the process by surface skimming and sedimentation tanks to an aeration technique at the rear of the grit chamber [51]. In actuality, during this stage, about 41% of MPs are dropped [52]. The concentrations of MPs in this investigation's effluent and influent were 79.9 MP/L and 47.4 MP/L, respectively. The effectiveness of the initial phase at a public treatment plant for wastewater in Glasgow, Scotland, was examined by Murphy et al. [53]. Average MPs decreased from 15.7 MP/L to 3.4 MP/L after this phase, with a removal rate of about 78%. A Spanish urban wastewater treatment plant's main stage saw a reduction of about 74% of MPs [54]. However, a high MPs removal rate (92%) was attained in the main stage of a sizable effluent treatment facility in Canada.

Most MPs were fibrous [30]. The majority of MPs, particularly those in the form of fiber were removed (99%) in this first stage in the study by Lares et al. with an input of 57.6 MP/L [25]. The remarkable efficiency attained in this study could be related to the fact that more than 96% of MPs are fibrous. The majority of MPs were successfully eliminated during this pretreatment phase, and the final stage can eliminate any that remain. However, to remove MPs, appropriate technology must be considered during the secondary or tertiary therapy stages. The main deposition and grit assembly steps make up the initial stage of the wastewater treatment process. MPs are mostly eliminated at this initial phase of the process by surface skimming and sedimentation. In actuality, during this stage, about 41% of the MPs are dropped [55]. In general, this preparation step worked well to get rid of MPs, and the rest can be taken care of in the next step. To completely get rid of MPs, though, you need to think about the right equipment during the second or third stage of treatment. It minimizes digester maintenance expenses by decreasing the need for frequent cleaning caused by the buildup of grit. It is advisable to prevent the accumulation of substantial deposits in pipelines and channels. Conversely, they are more prone to generating unpleasant smells and harmful organic substances. The control and maintenance of the aeration system will have an impact on many aspects of human resources. When compared to alternative methods of removing dirt, these technologies need greater energy resources.

#### 2.1.2 Primary settling

The majority of the suspended particles in sewage that is brought to the treatment facility are organic. These organic compounds in suspension increase the need for oxygen by bacteria in wastewater. In addition, suspended particles can disrupt biological activities, which would boost the demand for secondary treatment units and the oxygen required for aerobic processes. This would cause the secondary treatment system to need to be larger and cost more to run. Hence, it is imperative for wastewater treatment facilities to eliminate suspended organic particles, particularly when employing aerobic treatment methods for biological post-treatment.

Primary sedimentation tanks (PSTs) and primary clarifiers use gravity settling to remove suspended organic contaminants. Although PST can exhibit discrete or type I settling, the removal of suspended items is typically accomplished via flocculant or type II settling. The efficiency of the settling process is significantly impacted by the surface overflow rate, which is caused by the flocculant settling.

The suspended MPs' settable components were mostly eliminated by the principal settling process. Most of the suspended MPs that couldn't be sunk adhered to the flocs and deposited together, but some of them were skimmed off as scum [56]. Current research has suggested strategies for removing MPs via flocculation and primary settling, however, the characterization of microplastic byproducts and their physicochemical characteristics remains a challenge. The toxic effect of the chemicals produced during flocculation, as well as the effect of discharge time on primary discharge efficiency, is less well understood.

### 2.2 Secondary treatment

The wastewater's MPs content can reduce up to 0.2% of the total at the secondary clarifier, which is an adjacent circular-shaped assembly commonly next to biological treatment in WWTP. The remaining plastic particles will most likely be collected by sludge flocs or bacterial extracellular polymers in the aeration tank and then resolve in the secondary clarification tank. Because of protozoa or metazoan utilization, MPs may also become caught in sludge flocs. Additionally, chemicals used in the secondary treatment, such as ferric sulfate or other flocculating agents, may help eliminate MPs by causing dissolved small particles to collect and form the "floc" [57]. The interaction between microplastic and microbial or chemical flocs and the extent to which this can facilitate the removal of MPs are still unknown. Some MPs may also become caught in unstable flocs and fail to settle efficiently, causing these particles to move around dynamically in the aqueous phase and, as a result, avoiding separation during the settling stage [19].

The interaction duration of MPs with wastewater in the treatment train is another essential element for microplastic removal from secondary discharges. According to Carr et al., MPs with longer contact times are more likely to have surface biofilm coating [19]. These bio-coatings may operate as additives, changing the surface properties or relative concentrations of MPs [58]. Neutrally buoyant particles have a higher likelihood of eluding both skimming and settling techniques, such alterations could have a measurable impact on microplastic removal efficiency. In light of this, it may be worthwhile to look into how contact time and nutrient concentrations in wastewater affect the effectiveness of removing microplastic surface fouling. To better understand how biofilms form on MPs and how they affect particle transfer in freshwater and marine environments. The subsequent treatment, unlike the pre-treatment, eliminated more fragment particles than fibers. The studies showed that following the secondary treatment, the corresponding high amount of microplastic fragments dropped whereas the relative abundance of fibers enhanced [59]. One likely explanation is that during the pre-treatment, the easily settled or skimmed fibers were largely removed, while the remaining fibers may have possessed some inherent qualities, like neutral buoyancy, that made them impervious to further separation.

During secondary treatment, massive microplastic particles may be further eliminated, producing secondary pollutants with small concentrations of MPs. Microplastics larger than 500 mm were essentially non-existent in the secondary effluent, according to studies [26]. After secondary treatment, Talvitie et al. discovered that microparticles larger than 300 mm account for just 8% of the total [59]. Dris et al. discovered that following secondary treatment, MPs with diameters ranging from 500 mm to 1000 mm attributed to 43% of the total [47]. The cause for the elevated %age was unknown. Future research must concentrate on the particular microplastic removal capacity accomplished by different secondary treatment techniques in various operational contexts.

#### 2.2.1 Membrane bioreactor system (MBR)

The MBR stands for "membrane bioreactor," and it is a method of treating wastewater that combines biological treatment (aerobic, anaerobic) with membrane technology. In contrast to traditional biological treatments, which rely on a clarifier for gravity settling of sludge, this method employs microfiltration or ultrafiltration to achieve the same goal. The MBR process has many advantages over the more common activated sludge method. When comparing MBR with CAS, the solid retention time is longer in MBR whereas the hydraulic retention time is shorter.

**Fig. 3:**
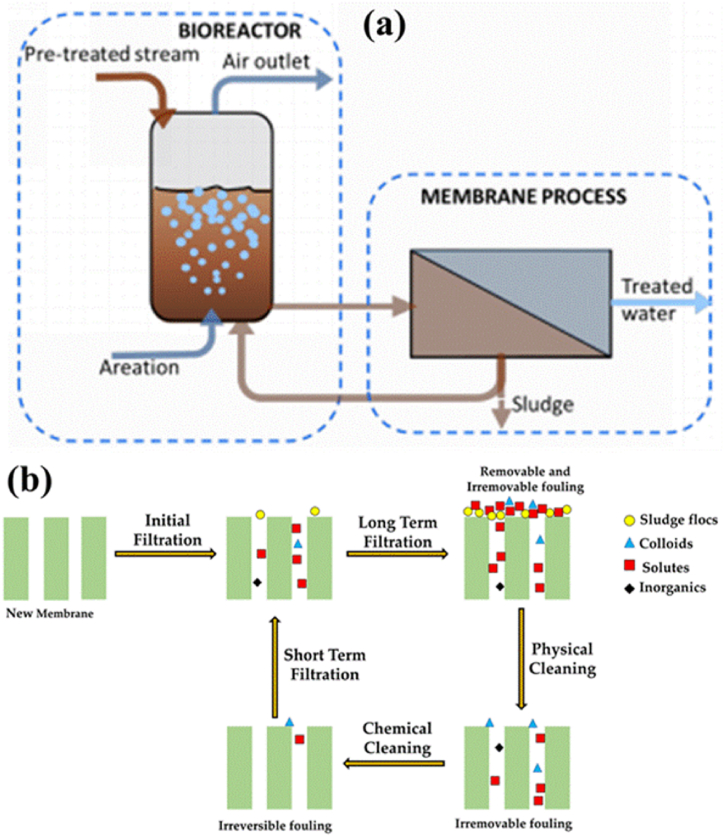
(a) Schematic diagram for the removal of microplastic using MBR technology. Reprinted with permission from Ref. [62] (b) Fouling development and removal by MBR. Reprinted with permission from [95].

The process of sludge separation is also improved by MBR as shown in Fig. 3 (a,b). Reduced space requirements and increased effluent quality due to MBR's lower biochemical oxygen demand, suspended particles, and turbidity. Additionally, MBR can be employed in anaerobic treatments by substituting up-flow anaerobic sludge blankets, expanded granular sludge beds, or anaerobic baffled tank reactors for standard anaerobic digestion. In order to create high-quality effluent with lower chemical oxygen demand than the conventional method, the anaerobic membrane bioreactor uses a biomass concentration control [13,14].The bioreactor system eliminated MPs primarily by microbe intake and the development of effluents. The accumulation of MPs in WWTPs was found to be accelerated by domesticated activated sludge. During subsequent secondary operation of settling, MPs containing sludge get eliminated [60].

The A2O bioreactor system is frequently used in WWTPs. It has a low microplastic removal efficacy due to sludge return. Some of the MPs (20%) that had been absorbed by the effluent would return to the aqueous phase. Moreover, microplastic breakdown in A2O was quite slow. Several functional microorganisms have been linked to a microplastic breakdown in previous research. The Rhodococcus genus of gram-positive bacteria was able to degrade 6.4% of the PP polymer mass in just 40 days [10]. Microplastics from PET film was destroyed by Ideonella sakaiensis in 6 weeks [61]. However, the current hydraulic retention duration in WWTPs (7–14 h) is insufficient for efficient microplastic breakdown by bacteria. That is why microplastic removal in WWTPs is not thought to be best accomplished using the traditional activated sludge method.

By biodegrading organic matter, MBR helps reduce the complexity of the solution, paving the way for MP purification and subsequent treatment [62]. Biodegradation of organic matter typically begins when pre-treated streams are introduced to the bioreactor. After the mixed liquor has been produced, it is pumped through a semi-crossflow filtration system to separate the components. The MP is concentrated in the retentate stream as a result of the membrane process.

Membrane bioreactor (MBR) technology has lately gained popularity in wastewater treatment plants. It's effective in getting rid of MPs because of the colloidal accumulation near its core (often between 6000 mg/L and 10,000 mg/L in liqueurs) (removal efficiency 99.9%) [59]. Membrane isolation and the conventional activated sludge process were merged by MBR technology. The MBR system's biofilm transmission side was able to retain the majority of the MPs. This suggested that the MBR system's capacity to eliminate MPs was significantly influenced by the adsorption effect. Furthermore, the size of MPs may have an impact on their clearance. The MBR system typically uses membranes with 0.1 m pore sizes. Microplastics might theoretically be eliminated in the MBR system [63]. In contrast to the conventional activated sludge process (CASP), this method is not only effective in treating and reusing wastewater, but it also takes up less room, makes less sludge, and is easy to scale up. Therefore, MBR has gained widespread recognition and has been successfully used in many various types of wastewater, particularly those containing developing contaminants including antibiotics, pesticides, personal care products, pharmaceuticals, and so on.

#### 2.2.2 Biofiltration

An advanced treatment unit was constructed using biofilter technology. The MPs had smaller particles and lower densities when they entered the biofilter treatment unit. This made removing MPs more challenging. The biofilter technique, on the other hand, had better microplastic extraction efficiency. The primary methods for removing MPs using biofilter technology were biofilm filtration and adsorption, which integrated physical and biological purification processes. The surface area of contact between the MPs and microbes increased as a result of the microbe film that was developing on the inert filter material coming into interaction with the MPs. Backwashing in the rising water flow easily eliminated excess microorganisms and retained MPs [56]. The microbial ecology and activity will be impacted by the presence of MPs because they are thought to be microbe transporters. According to Li et al., the addition of PVC swiftly changed the structure of the microbial community, and the number of living taxonomic units reduced from 1665 to 1533 [56]. This increased to 1735 functional taxonomic units. The proportion of each bacterium in the microbial strain altered a little over time.

As a result, the presence of PVC MPs had little effect on the change of the structure of the microbial community and did not cause a discernible decline in operational taxonomic units [46]. However, it is hopeful that virgin MPs have little impact on phosphorus-accumulating organisms, nitrite-oxidizing bacteria, or bacteria that produce ammonia. Therefore, it is important to avoid exaggerating the effect of plastic litter on the functionality of the bioreactor system. On the other hand, it remained unknown whether the microplastic additives were hazardous to microorganisms. Future research should take into account how microorganisms carrying MPs affect conventional pollution cleanup. Among these, more research is needed on how MPs affect microbe function after adhering to conventional pollutants. There are a lot of wastewater treatment processes that use biofiltration systems since they are popular, easy to operate, and energy efficient. How well biofiltration systems work is directly correlated to the microbiome's composition, community structure, and characteristics.

#### 2.2.3 Coagulation

**Fig. 4:**
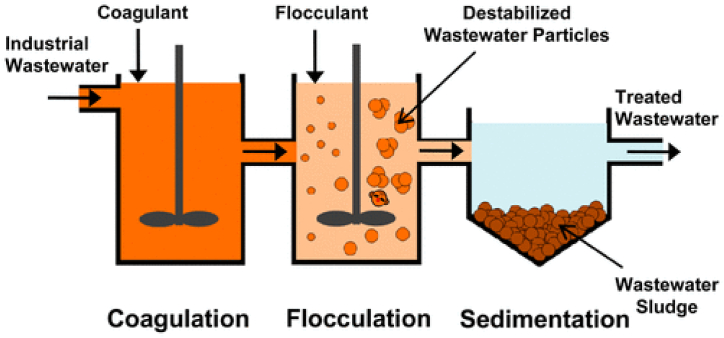
Schematic Diagram of Coagulation and Flocculation technology. Reprinted with permission from [70].

Coagulation was used to remove all large particles (>10 m) and 45%–75% of the smallest particles (5–10 m). Fibers can be removed more thoroughly (51–61%) than filaments or pellets because fibrous MPs were simpler to adhere to flocs than filament or pellet forms. In general, the coagulation/flocculation procedure can aid in the removal of up to 90% of MPs. Fig. 4 shows the schematic diagram of the working of the coagulation process with flocculation. The literature study revealed that this method was highly reliant on pH, MP size, shape, and components, as well as the quantity and type of coagulant and flocculant aids. There haven't been many studies on this method for MPs, especially for wastewater treatment systems. The future study must prioritize determining the proper coagulants/flocculant aids and their ideal circumstances for MPs removal and colloid removal [64].

Chemical coagulants such as aluminum salts and ferric and their derivatives were used in the initial step of tertiary treatment to destabilize surface charge and create floc with the MPs and other contaminants in the wastewater, which were subsequently removed by settling or skimming. The number of MPs had a noticeable impact on how MP flocs formed. Rezania et al. found a correlation between the removal of MPs and the coagulant dosage [65]. On the other hand, increasing the natural coagulant dosage is likely to reduce the rate of MP clearance. The zeta potential of MPs drops when the thrombotic dosage is increased too much, making MP floc formation more challenging.

Additionally, the coagulant used affects how well the coagulation process works. For instance, Polyethylene (PE), a waste material that is included in many wastes and has a larger percentage of MPs than the other categories, was investigated as a potential influence on coagulation factors based on aluminum and ferric anions [66]. This is why the aluminum coagulant is more effective at getting rid of PE. The effectiveness of clearing MPs from tiny PE rose from 8.3 to 36.9% when the aluminum coagulant dosage was increased from 13.5 mg/L to 405 mg/L (0.5 mm). In numerous studies, polyacrylamide (PAM) was discovered to help enhance coagulation effectiveness [67]. There have been multiple studies showing that coagulation mechanisms may effectively eliminate MPs from water [68,69]. Therefore, the coagulation technique is considered a good option for totally flushing MPs out of water whereas in recent years it has attracted a lot of attention from researchers because of the ease with which it may be used, its low cost, and the amount of energy it saves.

#### 2.2.4 Flocculation

Flocculation is an important part of treating water because it can remove suspended colloidal particles and other dissolved contaminants from water bodies so that solids and liquids can be separated and the water is cleaned [71,72]. Ultimately, the efficacy of flocculation depends heavily on the quality of the flocculants that are applied.

There are primarily three types of flocculants, including inorganic coagulants, synthetic organic polymeric flocculants, and composite flocculants. The most common inorganic coagulants used in water treatment facilities are poly aluminum chloride (PAC) or its polymer because of their low cost, low toxicity, and high availability. The creation of small flocs, the demand for high dosages, common removal efficiencies, and the ease of being affected by changes in water quality and pH conditions are some of the drawbacks associated with the use of these flocculants [73,74]. In the treatment of flocculation, the use of synthetic organic polymeric flocculants including polyethyleneimine, polyvinyl pyridinium salt, and polyacrylamide and its derivatives has been reported [75,76]. it has the potential to significantly reduce the demand for chemical oxygen as well as the need for biological oxygen.

#### 2.2.5 Dissolved air floatation (DAF)

The principle of DAF is that floc is brought to the water's surface when air bubbles are attached to it. Sludge is removed by skimming the floc that has settled on the top of the tank, often known as the "float". The water that has been purified is taken from the bottom and is sometimes referred to as the subnatant or the "floated" water. The flotation tanks, and often the flocculation tanks as well, need to be completely contained in a structure to prevent damage from the elements that may disrupt the float. The efficiency of coagulation and flocculation is crucial to the success of flotation as it is with other clarifying procedures. Dosing with polyelectrolytes is commonly used to make up for floc fragility or poor performance when the water temperature is low. Other clarity procedures are often better suited for treatment of directly abstracted waters, especially when the turbidity routinely reaches roughly 100 NTU, however the approach has been utilized effectively for certain such waters.

The DAF method is a three-stage procedure that involves the separation of solid particles (flakes) that are suspended in a liquid medium (water) through the action of microbubbles of gas (air). These air microbubbles cling to the flakes' surfaces, amplifying the force exerted on them and propelling them upward, where the sludge collects for some time before being collected by the proper mechanisms on the floatation tank's surface. When coagulants are added, DAF has the potential to be an effective method for MP removal from wastewater [59]. Studies evaluating the efficacy of DAF in removing MPs under various circumstances, such as MP density, size, shape, and composition, have not been conducted. As a result, it is now difficult to provide correct and thorough observations for this technology's elimination of MPs. This is an intriguing research gap that should be investigated further.

#### 2.2.6 Magnetic extraction

Magnetic extraction is a separation technique that uses magnetic fields. Because of their inexpensive cost, high specific surface area, and ease of use, they were chosen for this study. To make Fe nanoparticles hydrophobic, they were coated with hexadecyl trimethoxy silane permitting magnetic separation of MPs from water [77]. They were able to recover 92% of polyethylene and polystyrene beads with a diameter of 10–20 mm and 93% of microplastics with a diameter of more than 1 mm from saltwater by employing this method and a straightforward technique. A total of 84% and 78% of microparticles (MPs) with sizes ranging from 200 μm to 1 mm were retrieved from freshwater and sediments, respectively.

**Fig. 5:**
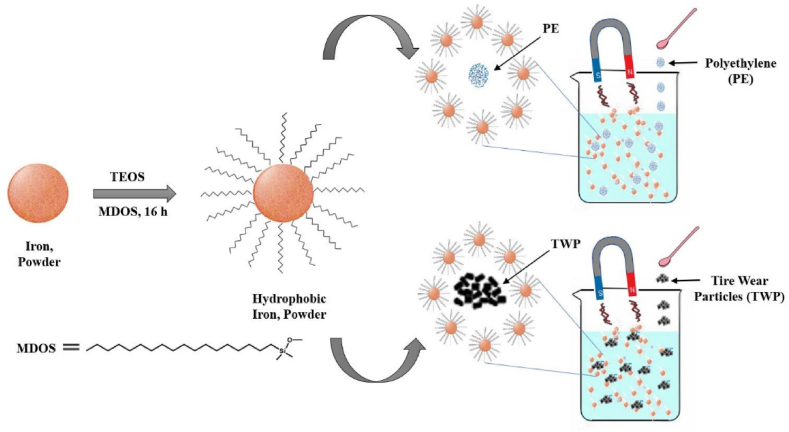
Mechanism of magnetic extraction technique. Reprinted with permission from [78].

The efficacy of magnetic extraction in eliminating tiny MPs was shown to be superior. The presence of soil particles hindered the collision between Fe nanoparticles and MPs, resulting in reduced recovery in sediments. Moreover, the presence of lipophilic compounds or biota in sediment samples will significantly restrict the impact due to nonspecific nanoparticle binding. As a result, the scientists concluded that this technology may be better suited to the treatment of drinking water. Fig. 5 shows that hydrophobic Fe-silane-based nanocomposite (Fe@SiO_2_/MDOS) was synthesized and applied to the separation of MPs from freshwater [78].

### 2.3 Tertiary system

The tertiary treatment may give significant extra polishing in the elimination of MPs. The percentage of MPs in the effluent was reduced from the influent to between 0.2% and 2% after tertiary treatment. The removal efficiency of MPs is determined by the treatment techniques used with membrane-based technologies exhibiting the best results. Talvitie et al. [59] examined the removal efficiency of various tertiary treatment procedures, including disc filter (DF), rapid sand filtration (RSF), dissolved air flotation (DAF) for secondary effluent treatment, and membrane bioreactor (MBR) for primary effluent treatment. They discovered that MBR had the highest removal effectiveness (99.9%), followed by RSF and DAF which had 97% and 95% removal efficiency, respectively. The efficacy of DF removal ranged from 40% to 98.5 %.

Similarly, in the evaluation of WWTPs in New York, two plants with membrane filters did not emit microbeads, although the other four plants with advanced filters (a quicksand filter, a continuous backwash filter, and two filters of unknown type). After the tertiary treatment, it was found that the specific needs fractions were the most prevalent [49]. When compared to the secondary effluent, the huge quantity of fiber in the final effluent may occasionally be higher. This might be a result of the easier longitudinal passage of filters or membranes through fibers. Therefore, procedures for the last stage are required to eliminate particular tiny and fiber-like MPs from the wastewater.

#### 2.3.1 Chlorination and the UV-oxidation

The most prevalent modern oxidation procedures in effluent treatment plants were chlorination and UV oxidation. In wastewater treatment plants, chlorine is a common disinfectant. The attack of chlorine on MPs was not entirely successful [79]. Because of microplastic breaking, the chlorination procedure enhanced their abundance [80]. Chlorine has the potential to break old ties while forming new ones. Because of its strong oxidizing tendency, chlorination altered the physical and chemical properties of MPs [81].

Even at high doses with longer exposure, the chemical bond change was barely perceptible [79]. Due to competitive interactions and chlorine biofilms, the effect of microplastic composition in chlorination may be changed. UV oxidation on the MPs' surface led to changes in their shape and the composition of chemicals [2]. Virgin-type MPs have fairly homogenous and compact textures. After UV oxidation, the textures of microplastic became fairly rough. Flakes or granular oxidation, cracks, and flakes were all general degradation scenarios for the PS, PE, and PP. On the other side, flakes and fractures in MPs made them easier to break, culminating in nanoscale and smaller plastics.

UltraViolet irradiation produces proxy free radicals which undergo subsequent reactions to create cross-link chemicals. And carbonyl group on the molecular chain will break to minimize Relative molecular mass. The intermediates and toxicity of Ultra Voilet oxidation MPs were unknown. In-depth research is needed to determine the impact of the Ultra Voilet irradiation period and the environmental variable on microplastic breakdown. Furthermore, the effect of the salinity and the dissolved organic on a microplastic breakdown in WWTPs must be considered. It is Simple, rapid, and efficient process. The release of volatile compounds and aromatic amines is one of the limitation.

#### 2.3.2 Ozonation

The polymer that forms MPs can be broken down by ozonation into the functional groups that have oxygen [82]. The ozone technique of treatment can change the physio-chemical characteristics of the polymers, including their close to the main, solubility, surface tension, and hydrophobic characteristics, as well as their decreased melting point and viscosity [83]. Both organic and non-organic pollutants, as well as a vital number of MPs, were oxidized using ozone technology. 90% of MPs were eliminated by ozonation after a 30-min processing period [84]. More than 90% of the microplastic dissolve after 1 h of ozone exposure at a temperature between 35° Celsius and 45° Celsius [82].

Because the ozonation method only reduced large-size MPs to smaller sizes, the output MPs concentration was only marginally higher than the input MPs concentration in some circumstances [85]. The expense of operation may be one issue limiting the usage of ozonation for MP removal. This approach required a significant dosage of ozone even though the rate of degradation increased noticeably in a shorter operating period. Additionally, if the treatment is not finished during ozonation, intermediate compounds that are bad for the environment and human health may form. The high running costs of ozonation plants could be a barrier to their widespread use for MP removal. This method needed a high ozone dosage, despite the fact that the deterioration rate rose drastically with decreasing working time. Further, if ozonation is not carried out thoroughly, harmful intermediate chemicals might be generated, endangering both human and environmental health.

#### 2.3.3 Membrane filtration

**Fig. 6:**
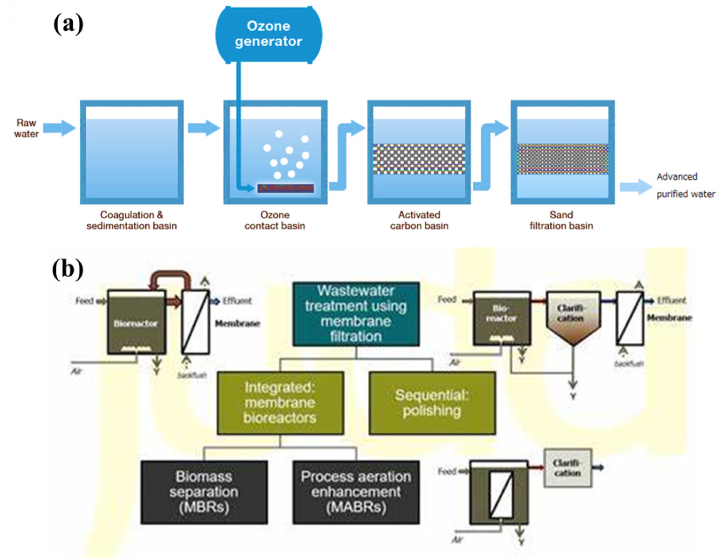
Schematic diagram of the removal technology (a) Ozonation (b) Membrane filtration

Membranes with homogeneous pores have been used widely in the treatment of wastewater. Different types of membrane filtering technologies were used to intercept MPs in the aqueous phase. The simple process schematic diagram can be seen in Fig. 6 (a,b). The researchers employed DF with 10-μm pore size to remove MPs in Daegu, South of Korea [94].The pore size of the ultrafiltration membrane was greater than the microplastic particle size (nanoscale). As a result, the ultrafiltration membranes fully rejected MPs [66]. Filtration of MPs resulted in a 38% reduction in final water flux [86]. This study depicts that there was an interaction between membrane holes and the surfaces and MPs. MPs were quickly adsorbed onto the membrane surface as well as inside and on top of the pores. As the exposure period rose, more MPs were passing through the barrier. Polysulfone membranes, for example, were more permeable than other membranes. Therefore, the attracting polar force was balanced by repelling electrostatic forces brought about by the membrane charge and the microplastic [86]. More investigation into competent and efficient techniques of cleaning is required to lessen the impact of MPs on membranes. There is a diverse selection of commercial membranes offered by many manufacturers, with a wide variety of applications and module configurations available. Efficient and expedient, even at elevated doses. The system generates a superior grade treated effluent. There are no specific chemical requirements. However, the expenses associated with investments are sometimes excessive for small and medium-sized enterprises. High energy demands. Membrane filtering systems can exhibit substantial variations in their design. The exorbitant expenses associated with maintenance and operation. The phenomenon of fast membrane clogging, also known as fouling, occurs when large quantities of substances accumulate on the membrane surface.

#### 2.3.4 Activated carbon filtration

Water purification with activated carbon filters is a common practice used to render water safe for disposal or for use in industrial operations by eliminating organic contaminants and/or extracting free chlorine. The formation of trihalomethanes, a class of recognized carcinogens, is prevented when organics like humic and fulvic acid are removed from drinking water.

Similar to other water purification techniques, Activated Carbon (AC) filtration can't guarantee a 100% clean supply of water. AC filtering is ineffective against some contaminants, like as salt, bacteria, fluoride, and nitrates. Filters designed for air conditioning systems are useless for softening water. Furthermore, activated carbon water treatment is often exclusively utilized in domestic point-of-use filters, and this type of treatment is ineffective against heavy metals such as lead.

**Fig. 7:**
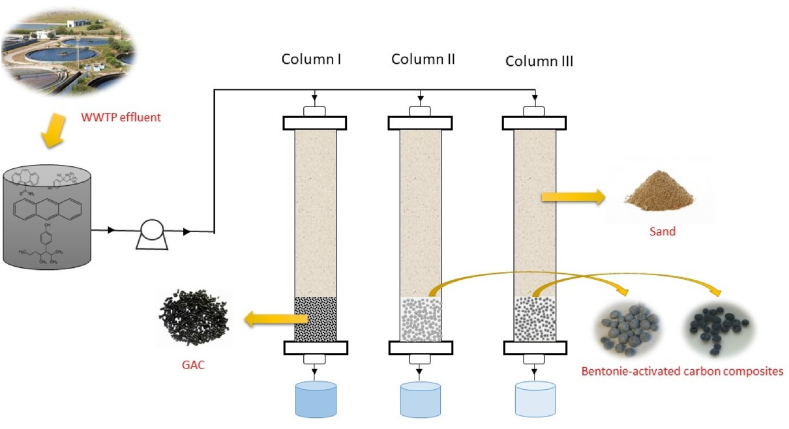
Schematic diagram of the GAC for the removal of micro pollutants from wastewater. Reprinted with permission from Ref. [88].

In previous years, a technique called GAC (granular activated carbons) filtering has been used to remove a variety of emerging pollutants from an aquatic environment. In a drinking treatment facility, Wang et al. looked into the GAC filtration system's capacity to eliminate MPs [87]. Up to 60.9% of MPs may be removed with this approach, which is less than other conventional techniques including flocculation or coagulation, sand filtration, RSF, and ozonation. Additionally, PE accounted for the majority of MPs expelled in the study when compared to PAM and PP. In the GAC method, pollutants are removed by a process that combines physical adsorption and biological decomposition. However, it is not yet known how to remove MPs from GAC. Filtering would therefore be a useful tool for getting rid of MPs at low concentrations. A cost-benefit study of various filtration rates and filter medium types would be interesting. Filtering would therefore be a useful tool for eliminating MPs in low quantities. A cost-benefit study of various filtration rates and filter medium types would be interesting. The filtration using granular bentonite-activated carbon composite (Ben-AC) was found beneficial to remove different micropollutants with varying physicochemical properties from WWTP effluent. The design of the filtration unit includes three columns packed with GAC, sand, and Ben-AC composite shown in Fig. 7 [88]. It requires low maintenance as compared to other process. The process of using filter is very easy. The efficiency rate is about 90% to reduce contaminants. Relatively high investment is required. The cost of materials is also high. The performance depends on the type of materials.

##### 2.3.4.1 Future perspective

At the moment, there is a lot of focus on getting rid of MPs, but the outcomes aren't great. Technology for eliminating MPs has a lot of space for development and improvement. It's possible that a single approach to removing MPs is wrong, but that a combination of two or more approaches yields surprising results. In the future, scientists may investigate the possibility of an organic integration between different kinds of technology. In spite of the fact that adsorption, membrane filtration, and chemically induced coagulation-inoculation-sedimentation are all capable of removing MPs with a high level of effectiveness, it can be challenging to implement these processes in real-world settings to treat significant quantities of wastewater. Bio-remediation techniques, on the other hand, can treat relatively large amounts of wastewater despite having a low removal effectiveness. In the future, it will be a difficult task to simultaneously attain high removal efficiencies and volumes at the same time. It's feasible that attention could be redirected to different types of organic substances. As an example, due to its naturally porous structure, wood is an excellent filter and adsorption material. Researchers are moving towards the synthetic fiber or plant fiber to make disposable plastic materials [[89], [90], [91], [92], [93]]. As a result, natural or functionalized wood as a substrate for membrane filtration and adsorption technologies is an option to examine for achieving savings and efficiencies. The technical removal of microplastics is not investigated thoroughly. Most important, the researcher should also focus on the nanosize plastic that form after the decomposition of microplastic using different ways as the small size could be a great threat. In the future, scientists will focus on developing methods for continuous microplastic removal at very high flow rates and low concentrations.

## 3 Conclusion

Several recent studies that looked at the transport of microplastic particles through wastewater treatment plants appear to confirm this. Removal of MPs at these facilities is very effective: 95–99% for all visible plastic sizes in the influent. These numbers are remarkable for treatment processes that were “not specifically designed” to target MPs.

These high removal efficiencies should give pause to growing demands for treatment plants to undergo immediate upgrades to reduce microplastic discharges. It should be noted that plastics make up only a minute fraction of solids in the influent stream and the existing solid removal processes, which make use of only basic physical processes, achieve removal efficiencies over 98% by utilizing only nominal density differences and gravity. It would therefore be difficult to justify significant expenditures to improve existing unit processes that already appear to remove over 98–99.9% of the plastics in influent streams. Demands to incorporate “advanced filtration” and other “innovative treatment trains” into plant design may appear to be a bit gratuitous, considering these removal percentages. The operational challenges resulting from any such modifications are likely to be daunting, not to mention costly. Incorporating filtering processes into operationally compliant units will result in minor increases in removal efficiency at best and unforeseen operational disturbances at worst. The latter will be especially noticeable if these modifications are sited at locations where the microplastic counts are highest. If such measures are adopted, plants will have to implement aggressive cleaning strategies to minimize flow restrictions and avoid other unforeseen operational impairments. These cleaning challenges and related maintenance issues would likely be costly and highly disruptive to routine plant functions.

Based on our knowledge of existing filtration technologies, most of the proposed modifications could be subject to extreme surface fouling and have unintended long-term operational consequences. Until such issues are fully vetted and addressed, it is prudent to utilize caution. Existing unit processes still appear to be suitable and compliant with the widest array of micro- and macro-hydrophobic residues including microplastic residues in wastewater effluents.

Currently, there is a lack of a comprehensive global strategy for remediating or reducing microplastics. One strategy would be to establish and implement long-term policy initiatives for the reduction of plastic waste, such as improved recycling and the adoption of biodegradable alternatives. Furthermore, it is uncommon for the world's governing authorities to engage in debate and discussion regarding the aforementioned problem. The development of a control plan will provide significant challenges due to the extensive influence that synthetic polymers exert on both human livelihoods and economic systems.

As a result, more research should be done on the subject of micro plastic contamination and its removal technologies, which is a growing hazard to both human and animal health. There should be joint collaboration between the industries and research institutes to further strengthen the policy. Furthermore, a more comprehensive understanding of the interactions between MPs and other contaminants is required. Additionally, it is crucial to develop a better understanding of the degradation process of MPs in the environment. Moreover, the research community would greatly benefit from the development and availability of standard reference materials. Environmental impact assessments should be conducted to evaluate the effects of emerging polymer composites. Lastly, global governing bodies should actively promote the development and discussion of remediation solutions for the issue of MPs.

In general, the matter pertaining to removal of microplastics from water is a multifaceted and urgent one that necessitates additional inquiry, financial support from the scientific community, and proactive measures. We can ensure the safety of species and their habitats for future generations by maintaining research into the effects of micro plastics and implementing appropriate management techniques.

## Funding

This research received no external funding.

## CRediT authorship contribution statement

**Muhammad Salman Nasir:** Writing – original draft, Methodology. **Ifrah Tahir:** Writing – original draft, Validation. **Ahsan Ali:** Formal analysis. **Iqra Ayub:** Writing – original draft, Investigation. **Abdul Nasir:** Formal analysis. **Naseem Abbas:** Writing – review & editing, Validation, Supervision, Methodology, Data curation. **Uzair Sajjad:** Formal analysis. **Khalid Hamid:** Funding acquisition, Formal analysis, Data curation.

## Declaration of competing interest

The authors declare that they have no known competing financial interests or personal relationships that could have appeared to influence the work reported in this paper.
